# Animal Models for COVID-19 Therapeutic Development: Where We Are and Where We Need to Go

**DOI:** 10.3389/fmicb.2022.907406

**Published:** 2022-06-24

**Authors:** Sihai Zhao, Jianglin Fan, Enqi Liu

**Affiliations:** ^1^Laboratory Animal Center, Health Science Center of Xi'an Jiaotong University, Xi'an, China; ^2^Institute of Molecular Virology, Health Science Center of Xi'an Jiaotong University, Xi'an, China; ^3^Department of Molecular Pathology, Faculty of Medicine, Graduate School of Medical Sciences, University of Yamanashi, Chuo, Japan

**Keywords:** animal models, COVID-19, therapeutics, vaccines, humanized mouse model

## Introduction

Severe acute respiratory syndrome coronavirus 2 (SARS-CoV-2) causes coronavirus disease 2019 (COVID-19), which has spread worldwide and reached a pandemic level within several months (Bedford et al., 2020; Li et al., 2020; Hu et al., 2021). Globally, scientists are trying to understand and defeat COVID-19 using animal studies to explore potential therapeutics. Vaccination remains the best choice to protect susceptible populations; therefore, many institutions and companies have developed vaccines against SARS-CoV-2 infection and many therapeutics are under clinical investigations. For these undertakings, it is essential to use suitable COVID-19 animal models (Caldera-Crespo et al., 2021; Munoz-Fontela et al., 2022).

## Essentials For Animal Models of COVID-19

Animal models are usually used in medical experimentation as an alternative to avoid potential hazards in humans (Liu and Fan, 2017). Appropriate COVID-19 animal models should be a very powerful tool in the study of the onset, development and culmination of this disease and provide helpful knowledge for its management. Making and choosing good models for COVID-19 requires taking full consideration of the following: (1) the animals should be susceptible to SARS-CoV-2, especially easily infected by the virus *via* the respiratory route; (2) the characteristics of the induced phenotypes or manifestations are similar to those of human COVID-19 patients, such as fever, coughing and myalgia or fatigue, pneumonia detected by computed tomography (CT) examination, along with sex- or age-related differences in severity (Huang et al., 2020); (3) immune responses should be similar to those in human coronavirus infections; and (4) the animals should have an appropriate cost. When testing vaccine protection effects, recovered infected animals should gain immunity to resist reinfection. These principles should be used for creating new models or evaluating candidate models for the development of COVID-19 therapeutics.

Due to the similarity of SARS-CoV-2 to other two beta coronaviruses, SARS-CoV-1 and Middle Eastern respiratory syndrome coronavirus (MERS-CoV), lessons from SARS-CoV-1 vaccine development can be helpful for accelerating SARS-CoV-2 vaccine development (Amanat and Krammer, 2020). Scientists have designed several SARS-CoV-2 vaccines to prevent COVID-19. Although the SARS-CoV-2 vaccine did not catch up with the first wave of the pandemic, the application of the vaccine has reduced the severe incidence of COVID-19 patients. Several faithful animal models for studying the pathogenesis of SARS-CoV-2 and its vaccine development have recently been created by scientists and have shed light on COVID-19 research. Here, we provide a brief review of these models, summarize what we have learned, and discuss their usefulness for testing the immunological response of vaccine injection and post-challenge safety.

## Non-Human Primate Models of COVID-19

Non-human primates (the rhesus macaque *or Macaca mulatta*, cynomolgus monkey, or *Macaca fascicularis*, common marmoset or *Callithrix jacchus* and African green monkeys or *Chlorocebus aethiops*) have been used to explore the pathogenesis and test the potential therapies of SARS-CoV-2 (Hartman et al., 2020; Lu et al., 2020; Rockx et al., 2020; Yu et al., 2020; Woolsey et al., 2021). All the above mentioned primates can be infected by experimental inoculation with SARS-CoV-2. The rhesus macaque is the most susceptible to SARS-CoV2 infection, followed by the cynomolgus monkey and the common marmoset (Lu et al., 2020). The viral replication state of nasopharyngeal swabs, anal swabs and lungs in old rhesus macaques was more active than that in young monkeys for 14 days after SARS-CoV-2 challenge (Yu et al., 2020). In SARS-CoV-2-infected macaques, viruses were discharged from the nose and throat in the absence of clinical symptoms and could be detected in both type I and II pneumocytes in the foci of diffuse alveolar damage and ciliated epithelial cells of the nasal, bronchial, and bronchiolar mucosae (Rockx et al., 2020). SARS-CoV-2 also causes pulmonary infiltrates in these monkeys, a hallmark of human disease shown in CT scans, which can be detected by chest radiographs in all infected rhesus macaques (Munster et al., 2020). Another important finding is that monkeys that have recovered from SARS-CoV-2 infection cannot be infected again in the near future (Yu et al., 2020). This finding also provided an important clue for antibody-mediated protection, suggesting that COVID-19 can be prevented through either natural infection or vaccination. Autopsy examinations revealed that the lungs, throat, bronchi, and spleen were infected in both rhesus monkeys and cynomolgus monkeys (Lu et al., 2020; Yu et al., 2020). These observations suggested that the rhesus monkey and cynomolgus monkey models can be used to evaluate vaccines and drugs to treat or prevent COVID-19. Gao et al. developed a purified inactivated SARS-CoV-2 virus vaccine candidate (PiCoVacc) and then conducted a challenge experiment in rhesus monkeys. They reported that vaccine immunization significantly reduced the pathological changes in the lungs of rhesus monkeys and the viral titers were also significantly decreased whereas no antibody-dependent enhancement was observed (Gao et al., 2020).

A recent report showed that African green monkey (AGM), another non-human primate, had robust SARS-CoV-2 replication and developed pronounced respiratory disease (Hartman et al., 2020; Woolsey et al., 2021). Shedding of SARS-CoV-2 from both respiratory and gastrointestinal tracts was also documented in AGMs, which may mimic human COVID-19 cases (Hartman et al., 2020). Therefore, AGM may provide another option for COVID-19 therapeutics and vaccine evaluation. These data suggest that monkey models are useful to evaluate vaccines and therapeutics for treating or preventing COVID-19. The advantages and disadvantages of non-human primates compared with those of other animal models are summarized in Table 1. However, high costs and ethical issues may strictly limit the widespread use of non-human primate models in most institutions.

**Table 1:** Comparison of animal models and human COVID-19 features.

	**Human**	**Primates**	**Mouse (hACE2 Tg)**	**Ferret**	**Hamster**
ACE2R similarity with human	Same	High	Low (wide-type)	High	High
Susceptibility	+	+	+	+	+
Respiratory transmission	+	+	+	+	+
Pneumonia	+	+	Mild	+	+
Severity differences (sex-related)	+	NR	NR	NR	NR
Severity differences (age-related)	+	+	NR	NR	NR
Resistant to reinfection	±	+	NR	NR	NR
Cost		Extremely high	Low	Middle	Low
Animal size		Big	Small	Middle	Small
Animal sources		Limited	Easy	Limited	Easy
Widely used		±	+	–	–
Reproductive cycle		Long	short	Long	short

*NR, not reported*.

## Rodent Models of COVID-19

The genetically manipulated mouse model is relatively cheap and easy to use (Korner et al., 2020). It has been reported that SARS-CoV-2 infection can occur through the angiotensin converting enzyme II receptor (ACE2R) for cellular entry (Zhou et al., 2020). Since 11 of the 29 amino acids in the critical region of the human ACE2R receptor are different from those of the mouse, SARS-CoV-2 is unable to naturally infect mice. Therefore, human ACE2R transgenic mice were developed and analyzed for potential application in the study of COVID-19 (Netland et al., 2008; Bao et al., 2020). Although hACE2R transgenic mice showed signs of weight loss and interstitial pneumonia after infection, the symptoms were mild and much different from those of humans (Bao et al., 2020). The hACE2R transgenic mice developed by Netland et al. also exhibited brain infection of SARS-CoV-2, which is rare in humans (Netland et al., 2008). These drawbacks may puzzle or mislead researchers for interpretation of their studies. The development of lung cell-specific expression of the hACE2R gene in transgenic or knock-in models without endogenous mouse ACE2R gene may help to broaden the use of mouse models in COVID-19 studies in the future. Except for modifying the mouse ACE2R for SARS-CoV-2 infecting host cells, another reverse genetics strategy was to design a recombinant mouse-adapted SARS-CoV-2, designated as SARS-CoV-2 MA, which can use mouse ACE2R for entry into cells (Dinnon et al., 2020; Leist et al., 2020). SARS-CoV-2 MA was reported to successfully infect and replicate in the upper and lower airways in both adult and aged BALB/c mice (Dinnon et al., 2020). SARS-CoV-2 MA caused more severe disease in aged mice, which resembles the human features of COVID-19, and exhibited more clinically relevant phenotypes than those seen in Hfh4-ACE2R transgenic mice (Dinnon et al., 2020). This model provided a powerful tool for the study of SARS-CoV-2 pathogenesis and has been used to evaluate vaccine and antiviral therapeutics performance (Leist et al., 2020).

As rats are unsusceptible to SARS-CoV-2, they are not used to study of COVID-19. However, Syrian hamster ACE2R proteins are reported to be highly similar to human ACE2R with 3–4 mutations at the interface (Chan et al., 2020). After being infected with SARS-CoV-2, hamsters showed weight loss, drowsiness, hairiness, hunched back, and shortness of breath. Olfactory and taste dysfunctions are common in mildly symptomatic COVID-19 patients. The food-searching behavioral test showed that anosmia may happen in SARS-CoV-2-infected hamsters, and the level of anosmia was associated with olfactory epithelium damage (Reyna et al., 2022). In addition, high titers of SARS-CoV-2 were found in the hamster lungs and intestines. The phenotypes of human upper and lower respiratory tract infections are very similar to those of hamsters, so this model benefits scientists who like to use small animals to perform COVID-19 research or therapeutic screening (Sia et al., 2020).

## Other Animal Models of COVID-19

Ferrets, another small animal, have special value in the research of virus-related diseases. After being infected with respiratory viruses, ferrets can show symptoms similar to those of humans. For example, ferrets infected with influenza virus can even sneeze like humans, and can easily transmit the virus to other ferrets (Herfst et al., 2012). SARS-CoV-2 can infect ferrets through the respiratory tract and can replicate in infected cells (Shi et al., 2020). The temperature of the infected ferrets rises slightly and infection does not cause serious illness or death. Although fatalities were not observed, SARS-CoV-2-infected ferrets shed the virus in nasal washes, saliva, urine, and feces up to 8 days post-infection (Kim et al., 2020). Ferrets give birth once per year, with 6–8 l, so they do not breed as rapidly as mice. To a certain extent, the slow reproduction speed may limit the wide application of ferrets. Similar to ferrets, farmed minks are also susceptible to coronaviruses. On farms, minks are infected with SARS-CoV-2 and can transmit it to humans (Enserink, 2020; Oreshkova et al., 2020; Sharun et al., 2021). Laboratory evidence also showed SARS-CoV-2 can be transmitted among minks and resulted severe respiratory syndrome (Shuai et al., 2021). Among middle- or large-sized animals, it was reported that SARS-CoV-2 can replicate efficiently in cats, with younger cats being more permissive and perhaps more importantly, the virus can transmit between cats *via* the airborne route (Shi et al., 2020). Dogs exhibit low susceptibility, whereas livestock, including pigs, chickens, and ducks, are not susceptible to SARS-CoV-2 (Shi et al., 2020). Previous reviews also have discussed the potentials of these animal species in COVID-19 studies (Munoz-Fontela et al., 2020; Sharun et al., 2020; Shou et al., 2021).

## Discussion And Perspectives

The currently reported COVID-19 animal models were summarized in Table 1. The advantages and disadvantages of the COVID-19 animal models that are currently available was discussed in this and previous study (Shou et al., 2021). To date, it seems that no animal model is perfect when evaluating potential drugs and vaccines (Munoz-Fontela et al., 2022). The most useful animal model is the rhesus macaque, but because of the substantial cost and specific facility requirements, the monkey model is unlikely to be widely used in common laboratories. The ferret model has special applicability in the study of respiratory viruses, but the low breeding rate and the lack of suitable facilities make it difficult for wide use in COVID-19 studies. The limitations of COVID-19 animal models are also apparent (Sharun et al., 2020). First, no model can fully mimic human COVID-19, and the findings of each model are needed to confirm with other animal models and to increase its credibility. Second, although the non-human primate model can better represent human COVID-19, it faces stricter ethical review and is not easy to be widely used. Mice are cheap and there are many genetically modified models available, but their immune system responses differ from those of humans. Humanized mouse models may become a feasible study tool for COVID-19 research in the future. At present, various genetically engineered mouse models are currently on the way, and hopefully, these new models will provide a unique research tool to fight against COVID-19 in the future.

## Author Contributions

JF and EL designed this project. SZ wrote the paper. All authors contributed to the article and approved the submitted version.

## Funding

This study was supported by grants from the Natural Science Foundation of Shaanxi Province (Grant Nos. 2020PT-004 and 2020PT-001).

## Conflict of Interest

The authors declare that the research was conducted in the absence of any commercial or financial relationships that could be construed as a potential conflict of interest.

## Publisher's Note

All claims expressed in this article are solely those of the authors and do not necessarily represent those of their affiliated organizations, or those of the publisher, the editors and the reviewers. Any product that may be evaluated in this article, or claim that may be made by its manufacturer, is not guaranteed or endorsed by the publisher.
